# Chatbot-delivered mental health support: Attitudes and utilization in a sample of U.S. college students

**DOI:** 10.1177/20552076241313401

**Published:** 2025-01-17

**Authors:** Gavin N. Rackoff, Zhenyu Z. Zhang, Michelle G. Newman

**Affiliations:** Department of Psychology, 8082 The Pennsylvania State University, University Park, PA, USA

**Keywords:** Chatbots, mental health, artificial intelligence, health services, utilization, survey research

## Abstract

**Objective:**

Chatbots’ rapid advancements raise the possibility that they can be used to deliver mental health support. However, public utilization of and opinions toward chatbots for mental health support are poorly understood.

**Methods:**

Survey study of 428 U.S. university students who participated in early 2024, just over one year after the release of ChatGPT. Descriptive analyses examined utilization of and attitudes toward both traditional mental health services (i.e. psychotherapy, counseling, or medication) and chatbot-delivered mental health support.

**Results:**

Nearly half (49%) of participants reported having used a chatbot for any purpose, yet only 5% reported seeking mental health support from a chatbot (8% when only considering participants with probable depression or generalized anxiety disorder). Attitudes toward traditional mental health services were broadly positive, and attitudes toward chatbot-delivered support were neutral and significantly less positive (*d *= 1.18, *p *< .001). Participants reported lack of need and doubts about helpfulness as barriers to using chatbot-delivered support more frequently than they reported them as barriers to traditional services. Cost, time, and stigma barriers were less frequently reported for chatbot-delivered support than for traditional services. Attitudes were generally consistent as a function of mental health status.

**Conclusion:**

Among U.S. students, utilization of chatbots for mental health support is uncommon. Chatbots are perceived as less likely to be beneficial, yet also less affected by cost, time, and stigma barriers than traditional services. Rigorous outcome research may increase public trust in and utilization of chatbots for mental health support.

## Introduction

Chatbots, which are computer programs designed to simulate conversation with human users,^1^ have become increasingly sophisticated in their ability to perform intelligent, human-like communication. These technological developments are reflected in consumer-facing generative artificial intelligence chatbots including ChatGPT^2^ and its successors as well as other paid and freely available chatbots. The rapid advancements in chatbots raise the possibility that they might be used to provide forms of support typically delivered by mental health professionals such as counselors and psychotherapists. Several commercial enterprises offer specialized chatbots designed for mental health concerns,^3^ and a case report suggested that some people had consulted general-purpose chatbots such as ChatGPT regarding mental health concerns.^4^ Systematic reviews have supported the usability and efficacy of chatbots for the delivery of some forms of mental health support.^5,6^ However, this literature has focused almost exclusively on research trial settings, in which participants have been given access to chatbots and subsequently assessed for their mental health symptoms (e.g. depression, anxiety, distress) and experiences using them. In order for chatbot technologies to deliver a public health impact, they must be adopted by consumers outside of trial settings. Highlighting the importance of this issue, one recent study found that only 3% of distressed persons invited to participate in a trial evaluating a mental health chatbot enrolled in the trial, raising questions about public adoption of mental health chatbots.^7^ Understanding real-world utilization of and public attitudes toward chatbot-delivered mental health support will help identify opportunities and barriers for implementation.

A small literature has examined attitudes toward and utilization of chatbots for mental health support. A survey of UK university students indicated that 47% had used a chatbot for any purpose, and 37% said they would feel comfortable talking with a chatbot about their mental health.^8^ Notably, this paper was published prior to the November 2022 public release of ChatGPT, potentially reducing its relevance to current technologies. Additionally, data on chatbot mental health support-seeking behavior were not reported. A study of South African university students found that 4.5% reported having used a chatbot for mental health support, and 35.2% viewed themselves as either “somewhat” or “very” likely to do so in the future.^9^ This survey was conducted between November and December 2022, comprising a mix of datapoints from before and after the public release of ChatGPT and calling for a more updated investigation. Finally, a study of U.S. adults screened for depression or anxiety symptoms indicated that 47% used “automated e-health tools for anxiety and depression” (p. 724), which encompassed chatbots, and those who had not used such tools agreed on average (mean score of 4.67 on a 1–7 scale) with a measure of intention to use them.^10^ This markedly high rate of reported use could mean that persons with anxiety or depression symptoms use chatbots for mental health support more often than participants in general population studies, and it calls for more research comparing chatbot support seeking as a function of mental health status. However, it is also possible that the study's broad definition of automated e-health tools led to overinclusion of users of non-chatbot digital self-help tools. Moreover, the data were collected prior to the release of ChatGPT, suggesting a need for an updated examination of rates of chatbot-delivered mental health support-seeking among both healthy and anxious or depressed participants.

Existing research leaves open several questions about the public's use of and attitudes toward chatbots for mental health support. Only one study directly assessed whether participants had utilized chatbots for mental health support, and the 4.5% utilization^9^ was low relative to estimated rates of perceived comfort (37%)^8^ and future likelihood (35.2%)^9^ of utilization. Because the most recent study took place within one month of ChatGPT's release,^9^ the gap between respondents’ past use and attitudes toward future use of chatbots could reflect changes in public sentiment, indicating a need for updated descriptive data on chatbot usage behaviors. Importantly, no prior study has compared attitudes toward chatbot-delivered mental health support to attitudes toward traditional services. Such comparisons would be valuable to contextualize the potential role of chatbots among existing services such as psychotherapy and psychotropic medication. Specifically, it might be possible to identify shortcomings in the traditional mental healthcare infrastructure that are better addressed by chatbots (e.g. cost and ease of accessing care), as well as areas in which consumer sentiment toward chatbots lags behind traditional mental health services (e.g. perceived helpfulness). Notably, two of the prior studies examining attitudes toward and utilization of chatbot-delivered mental health support examined college student samples.^8,9^ College students tend to report high levels of mental health concerns such as depression and anxiety^11^ and high utilization of mental health services such as psychotherapy and psychotropic medication.^12^ Therefore, college students comprise an outsize and especially important segment of the population that is likely to need and use mental health services. Moreover, examining college students, who tend to be early adopters of digital technologies,^13^ could provide a glimpse of attitudes that might be held by the broader public in the near future. Neither of the two prior studies of college students provided data on students’ depression and anxiety. Given the high prevalence of depression and anxiety among college students,^11^ it would be especially valuable to clarify attitudes and behaviors among students with and without elevated levels of these mental health symptoms.

The present study aimed to address the need for up-to-date data on how college students utilize and perceive chatbots for mental health support. The study also aimed to address the need for understanding these attitudes and behaviors in the context of students’ mental health concerns and other available mental health services. U.S. university students were surveyed in early 2024, just over one year after the public release of ChatGPT, allowing an indication of students’ initial utilization of current generative artificial intelligence chatbot technologies. We administered measures of depression and generalized anxiety disorder symptoms, enabling descriptive analyses focused on individuals who were likely to have a need for mental health services. Additionally, we measured traditional mental health service utilization and attitudes, allowing direct comparisons between chatbot-delivered and traditional mental health support and the identification of possible strengths and shortcomings of chatbots relative to other available services. The study aims to address the need for comprehensive and up-to-date information on chatbots’ current and possible future role in providing mental health support.

## Method

### Participants

Participants were 428 students at a public university in the northeastern United States. For information about the nature of the sample, please see demographic characteristics in Table 1. As can be seen in Table 1, the vast majority of participants were in the traditional college age range of 18–21 years (94%), and the sample was predominantly White (77%) and non-Hispanic (89%). Just under two-thirds (64%) of participants identified as female, and just over one-third (35%) of participants had probable depression or generalized anxiety disorder.

**Table 1.** Sample characteristics.

Characteristic	*N*	%
Gender		
Male	149	35
Female	273	64
Gender minority	6	1
Race		
White	328	77
Black or African American	45	11
Asian	35	8
American Indian or Alaskan Native	1	<1
Native Hawaiian or other Pacific Islander	1	<1
Unknown or do not wish to disclose	18	4
Ethnicity		
Hispanic	48	11
Non-Hispanic	380	89
Age		
18–21	404	94
22–35	24	6
Mental health status		
Neither depression nor generalized anxiety	275	65
Probable depression	46	11
Probable generalized anxiety	35	8
Probable depression and generalized anxiety	65	15

*Note. N *= 428.

### Procedures

Participants completed a survey in which they reported on their utilization of and attitudes toward traditional mental health services (i.e. psychotherapy, counseling, or medication). They then completed a nearly identical set of questions, in which the wording was modified to refer to “chatbots or digital conversational agents.” This section of questions was preceded by an introduction reading, “The next set of questions asks about your experiences and attitudes toward receiving mental health support from chatbots or digital conversation agents (e.g. ChatGPT).” Participants also completed measures of demographics, depression and anxiety, and other psychological constructs for other research not reported here. The survey was administered online via Qualtrics survey software between February and April 2024, and participants accessed the survey from their personal computers at the times and locations of their choosing. The entire survey took approximately 45 min to complete. All participants provided informed consent to participate. Participants were shown a consent form at the start of the survey with information about the study, and they were asked to indicate their consent to participate by checking a box reading “I agree to participate,” which appeared after the consent form. This method of obtaining informed consent and all study procedures were approved by the authors’ Institutional Review Board. Where applicable, we obtained all necessary copyright permissions to use survey measures. Participants received course credit for their participation.

### Measures

#### Utilization of traditional mental health services and chatbots

As a measure of mental health service utilization, participants were asked, “In the past 12 months, have you received any treatment for emotional or mental health problems (e.g. therapy, counseling, medication)?” As a measure of general chatbot utilization, participants were asked “Have you ever used a chatbot or digital conversational agent for any purpose (even if unrelated to mental health)?” As a measure of mental health support seeking from chatbots, we asked, “In the past 12 months, have you sought mental health support from a chatbot or digital conversational agent?”

#### Perceived qualities of traditional and chatbot-delivered mental health support

Participants completed the Mental Help Seeking Attitudes Scale (MHSAS), a self-report measure using bipolar adjective ratings to assess how respondents perceive mental health services such as psychiatry and counseling.^14^ This measure previously demonstrated strong factorial validity, convergent validity, and measurement invariance across important demographic (e.g. gender) and psychological (e.g. mental distress) variables.^14^ The questionnaire provided the stem statement, “If I had a mental health concern, seeking help from a mental health professional would be…” followed by nine adjective pairs (e.g. “useless vs. useful,” “unhealthy vs. healthy”), rated on a 7-point dimensional scale. Higher scores indicated more favorable attitudes toward seeking help. The MHSAS was administered as developed by the scale authors to assess the perceived qualities of traditional mental health services.^14^ Additionally, a modified MHSAS with the stem statement, “If I had a mental health concern, seeking help from a chatbot or digital conversational agent would be…” was administered to assess perceived qualities of chatbot-delivered mental health support. Both versions of the measure had excellent internal consistency (Cronbach's alpha = .92). The modified MHSAS adapted for the present study can be found in Supplementary Materials.

#### Barriers toward traditional and chatbot-delivered mental health support

We administered a modified version of the measure of structural barriers toward mental health service utilization as reported by Van Doren et al.^15^ This measure has not undergone formal validation but was previously used in a large national survey of college students.^15^ For participants who denied receiving traditional mental health services, we asked, “Which of the following explain why you have not received treatment for your mental or emotional health?” For participants who denied seeking mental health support from a chatbot, we asked, “Which of the following explain why you have not sought mental health support from a chatbot or digital conversational agent?” Response options included (1) “No need for services,” (2) “Financial reasons (too expensive, not covered by insurance),” (3) “Not enough time,” (4) “Don’t think [treatment/a chatbot or conversational agent] would help,” (5) “Concerns about mental health stigma,” and (6) Privacy concerns. Participants could select multiple options. Because this measure was modified for the present study, we have provided a copy in Supplementary Materials.

### Depression

We administered the Beck Depression Inventory, a 21-item self-report measure of depression severity.^16^ Psychometric investigations have supported this measure's convergent and divergent validity.^17^ Sum scores were calculated, and probable clinical depression was indicated by a score of 20 or higher, a cutoff found to possess 76% accuracy in screening for major depression.^18^ Cronbach's alpha in the present sample was .94.

### Anxiety

We administered the Generalized Anxiety Disorder Questionnaire-IV, a validated measure designed to screen for generalized anxiety disorder.^19^ Participants were identified as having probable generalized anxiety disorder if they endorsed all symptoms of the disorder as outlined in the *Diagnostic and Statistical Manual of Mental Disorders, 5th edition*.^20^ This measure possessed 83% sensitivity and 89% specificity for the identification of generalized anxiety disorder in addition to strong convergent validity with continuous anxiety measures.^19^

### Data analyses

We analyzed descriptive statistics on all measures to characterize utilization of and attitudes toward traditional and chatbot-delivered mental health support. Given that chatbot support seeking might differ as a function of mental health status, we also calculated utilization and attitude descriptive statistics separately for participants with versus without probable depression or generalized anxiety disorder and compared them with logistic regression. Participants with probable depression and/or generalized anxiety disorder were grouped together in these analyses because these concerns were highly comorbid in the sample and either mental health condition might be cause for seeking mental health support. Subsequently, to directly compare attitudes toward traditional and chatbot-delivered support, we used the *lme4* package^21^ in *R* to fit (1) linear mixed effect models comparing MHSAS scores among *all* participants and (2) logistic mixed effect models comparing rates of reported barriers among participants reporting *neither* use of traditional services nor seeking of chatbot-delivered support. All models incorporated a random intercept to accommodate repeated measures. Minor missing data (*n *= 11, 3%, had missing data on the MHSAS, and no participants had missing data on barriers) were handled using full information maximum likelihood estimation. To examine whether attitudes differed as a function of mental health status, we subsequently augmented these models with a simple effect and an interaction term involving depression or anxiety status. The interaction indicated if participants’ comparative attitudes toward chatbot-delivered support (vs. traditional services) differed as a function of mental health status. For linear models, Cohen's *d* was calculated by dividing the mean difference between the attitude measures (traditional vs. chatbot-delivered support) by the pooled standard deviation for repeated measures, as defined in equation (8) of Lakens.^22^ For logistic models, Cohen's *d* was calculated as $\frac{2\left(\frac{Z}{\sqrt{N}}\right)}{\sqrt{1-{\left(\frac{Z}{\sqrt{N}}\right)}^{2}}}$ .^23^ Cohen's *d* of 0.2, 0.5, and 0.8 were interpreted as small, medium, and large, respectively.

## Results

Descriptive statistics on the utilization of traditional mental health services, chatbots in general, and chatbots for mental health support are shown in Table 2. Within the full sample, 31% reported receiving traditional services in the previous 12 months, 49% reported using a chatbot, and 5% reported seeking mental health support from a chatbot. Among participants with probable depression or anxiety, rates of traditional service utilization (*B *= 1.57, *SE *= 0.23, *Z *= 6.96, *p *< .001, *d *= 0.71) and chatbot-delivered support utilization (*B *= 1.23, *SE *= 0.48, *Z *= 2.53, *p *= .011, *d *= 0.25) were significantly higher than among participants without probable depression or anxiety. Rates of traditional service utilization were significantly higher than utilization of chatbot-delivered support in both subgroups (no depression or anxiety: *B *= 2.21, *SE *= 0.41, *Z *= 5.37, *p *< .001, *d *= 0.54; probable depression anxiety: *B *= 2.55, *SE *= 0.34, *Z *= 7.41, *p *< .001, *d *= 0.78). Rates of utilization of chatbots for any purpose did not differ as a function of mental health status (*B *= 0.36, *SE *= 0.21, *Z *= 1.75, *p *= .080, *d *= 0.17). Thus, chatbot-delivered mental health support seeking was uncommon and less common than utilization of traditional mental health services, irrespective of mental health status.

**Table 2.** Utilization and attitude descriptive statistics.

Measure	Full sample		Without depression or anxiety		With depression or anxiety	
	*N*	%	*N*	%	*N*	%
Utilization						
Traditional mental health services	133	31	53	19	78	53
Chatbot (any purpose)	211	49	126	46	80	55
Chatbot-delivered mental health support	20	5	7	3	12	8
MHSAS score	*M*	*SD*	*M*	*SD*	*M*	*SD*
Traditional mental health services	5.78	1.08	5.81	1.05	5.72	1.15
Chatbot-delivered mental health support	3.40	1.18	3.42	1.22	3.36	1.12
Barrier						
No need for services						
Traditional mental health services	182	65	157	72	24	39
Chatbot-delivered mental health support	202	72	170	78	30	49
Don't think it would help						
Traditional mental health services	23	8	15	7	7	11
Chatbot-delivered mental health support	74	26	49	23	23	38
Financial reasons						
Traditional mental health services	30	11	18	8	12	20
Chatbot-delivered mental health support	6	2	3	1	3	5
Not enough time						
Traditional mental health services	59	21	38	18	21	34
Chatbot-delivered mental health support	19	7	8	4	11	18
Stigma concerns						
Traditional mental health services	17	6	9	4	7	11
Chatbot-delivered mental health support	7	2	3	1	3	5
Privacy concerns						
Traditional mental health services	18	6	11	5	7	11
Chatbot-delivered mental health support	21	7	10	5	10	16

*Note. N *= 428 for utilization and MHSAS descriptive statistics (of whom 146 had probable depression or anxiety); *N *= 282 for barrier descriptive statistics (of whom 61 had probable depression or anxiety). MHSAS and barrier questions used identical wording to assess attitude dimensions and barriers with reference to traditional services and chatbot-delivered support.

Please see Table 2 for descriptive statistics on the attitude measures. The average MHSAS scores indicated broadly positive perceptions of traditional mental health services and neutral perceptions of chatbot-delivered mental health support. The model indicated significantly more positive perceptions of traditional mental health services, *B *= 2.38, *SE *= 0.07, *t*(424) = 32.43, *p *< .001, *d *= 1.18. The augmented clinical status model indicated no interaction with mental health status, *B *= 0.03, *SE *= 0.16, *t*(418) = 0.17, *p *= .862, *d *= 0.02. Thus, irrespective of mental health status, participants perceived more possible benefits from receiving traditional mental health services than from chatbot-delivered mental health support.

Please see Table 3 for results from the models examining reasons for not receiving support among participants who denied both receiving traditional mental health services and seeking support from a chatbot (*n *= 282, of whom 61 had probable anxiety or depression). Barriers more commonly reported as reasons for not seeking chatbot-delivered mental health support (vs. receiving traditional services) were (1) no need for services and (2) not thinking the service would help. Thus, compared to traditional services, chatbot-delivered mental health support appeared more affected by barriers related to the perceived benefits of the service. Barriers more commonly reported as reasons for not receiving traditional services (vs. chatbot-delivered mental health support) were (1) financial reasons, (2) not enough time, and (3) concerns about mental health stigma. The only interaction involving mental health status occurred with the barrier of “not enough time.” This barrier was less strongly associated with traditional services versus chatbot-delivered support among participants with probable depression or anxiety, compared to healthy participants. Nonetheless, even among participants with probable depression or anxiety, chatbots were still less strongly associated with this barrier than traditional services (simple slope *B *= −5.51, *SE *= −1.52, *Z *= −3.64, *p *< .001, *d *= −0.44); this effect was merely smaller than among participants without depression or anxiety (simple slope *B *= −9.46, *SE *= −1.17, *Z *= −8.08, *p *< .001, *d *= −1.10). Thus, compared to traditional services, chatbot-delivered mental health support appeared less affected by barriers related to ease of access and stigma. Privacy concerns were not more strongly associated with either type of support. Participants therefore demonstrated different attitudes about reasons for not receiving traditional and chatbot-delivered mental health support, and these attitudes were generally consistent as a function of mental health status.

**Table 3.** Results of barriers to utilization models.

Term	*B*	*SE*	*Z*	*p*	*d*
Traditional vs. chatbot-delivered support					
No need for services	1.97	0.54	3.61	<.001	0.44
Don't think it would help	1.73	0.33	5.18	<.001	0.65
Financial reasons	−9.30	1.23	−7.54	<.001	−1.01
Not enough time	−8.23	0.91	−9.08	<.001	−1.29
Stigma concerns	−7.27	1.62	−4.50	<.001	−0.56
Privacy concerns	0.82	0.76	1.07	0.283	−0.13
Interaction with clinical status					
No need for services	−0.18	1.06	−0.17	0.867	−0.02
Don't think it would help	0.36	0.64	0.55	0.581	0.07
Financial reasons	0.54	1.37	0.40	0.691	0.05
Not enough time	3.95	1.64	2.40	0.016	0.29
Stigma concerns	−2.73	2.19	−1.25	0.212	−0.15
Privacy concerns	2.77	1.80	1.54	0.123	0.18

*Note*. *N *= 282 participants who reported neither using traditional services nor seeking support from chatbots. First set of terms (traditional vs. chatbot-delivered support) indicates degree to which a given barrier was more (or less) frequently reported for chatbot-delivered services (with traditional services as the reference group). Second set of terms (interaction with clinical status) indicates the degree to which such differences were more (or less) pronounced among participants with probable depression or generalized anxiety (with nonclinical participants as the reference group).

## Discussion

The present study is one of the first to investigate the utilization of and attitudes toward chatbot-delivered mental health support since the public release of ChatGPT, adding to the small body of survey studies examining these topics in adult samples from around the world.^8–10^ Converging with the findings of Gbollie et al.,^9^ who found that 4.5% of their sample had ever used a mental health chatbot, we found a similar percentage of participants who reported seeking mental health support from a chatbot in the past 12 months (5%). Moreover, the 8% rate of chatbot-delivered mental health support seeking among depressed or anxious participants in the present study was much lower than in a prior study of adults with elevated depression or anxiety symptoms (47%).^10^ This difference might be attributed to assessment methods, given that the present study specifically asked about the utilization of chatbots, rather than automated e-health tools in general. Thus, even among mentally distressed participants, chatbot-delivered mental health support seeking appears uncommon.

The analyses of attitudes provide possible explanations for the low rate of chatbot mental health support utilization. Participants endorsed positive views of traditional mental health services on average, yet they endorsed more neutral views of chatbot-delivered mental health support. Furthermore, participants were more likely to report a lack of need and perceived unhelpfulness as reasons for not seeking support from chatbots, and this pattern held irrespective of participants’ mental health status. In fact, over one-third (38%) of participants with probable depression or generalized anxiety disorder who did not receive chatbot-delivered support reported believing such support would not help. Thus, participants appeared wary of the potential utility of receiving support from a chatbot. Low chatbot utilization in the present sample might therefore be partially attributed to beliefs that chatbots would not be helpful. Given some promising findings for the acceptability and efficacy of mental health chatbot interventions,^5,6^ the public health impact of these interventions might be improved by disseminating information about their helpfulness to the population.

Attitude data also identified several perceived shortcomings of traditional mental health services that could be addressed by chatbots. Participants reported financial, time, and stigma barriers as reasons for not receiving traditional services more commonly than they reported as reasons for not seeking chatbot-delivered mental health support. All of these patterns were consistent as a function of mental health status, though chatbots’ relative advantage in terms of time concerns was somewhat smaller (though still significant) among participants with probable depression or generalized anxiety disorder. Thus, public opinion of chatbot-delivered mental health support might be more positive in terms of ease of access and stigma. Participants might perceive chatbots as more easy to access than traditional services given that many sophisticated chatbots, including some dedicated mental health chatbots, are currently available for free and can be accessed at any time via an internet-enabled device.^3^ The lower rate of stigma concerns for chatbot-delivered mental health support might have arisen due to the ability to access a chatbot discreetly from an internet-enabled device or the freedom from judgment one might perceive speaking to a nonhuman conversational agent versus a human service provider. Indeed, one study of user reviews of mental health chatbots found that some users appreciated the “judgment-free” experience of sharing concerns with a bot.^3^ Thus, for providers of chatbot-delivered mental health support, appealing to people's preferences for ease of use and nonjudgmental support might increase uptake. Of note, consumers may be rightfully skeptical of chatbots for mental health support, given the potential for generative chatbots to provide misleading or harmful information.^24^ For providers of mental health chatbots, establishing helpfulness and safety is necessary to provide a beneficial public health effect.

A major implication of the present study's findings is the need for high-quality research about the efficacy and safety of chatbots for mental health support. Traditional mental health services, which were viewed more positively and used more widely among participants in the present study, have been subjected to years of outcome research in clinical trials. This body of research has supported health care systems’ delivery and third-party reimbursement of traditional mental health services.^25^ Thus, the research supporting traditional mental health services’ efficacy and safety is a major reason that people receive these services when seeking care. Establishing the efficacy and safety of chatbots for mental health support is paramount to justify their implementation within health care systems and utilization by individual consumers. Of particular importance is the need to clarify which mental health concerns (e.g. depression, anxiety, and eating disorders) are most amenable to chatbot-delivered interventions and which chatbot-delivered interventions are most efficacious. In the present study, over half of participants with elevated depression or anxiety reported receiving traditional services, so it would also be valuable to investigate the possible utility of chatbot-delivered support as an adjunctive intervention among participants already receiving psychotherapy or medication.

Several limitations of this study deserve note. First, although the student sample enabled studying a possible early adopter population that comprises an outsize proportion of mental health support seekers, it precluded drawing conclusions to the general public. Therefore, replication in representative community samples is necessary. Second, the survey did not differentiate between general chatbots and dedicated mental health chatbots. Future research should examine both the extent to which people consult with general-purpose chatbots about their mental concerns and the extent to which people use chatbots designed to discuss mental health concerns. Additionally, although the modified version of the MHSAS administered in this study was based on the validated MHSAS, and the barriers to care measure aligns closely with previously used measures to assess barriers to care, neither of these measures as administered in the present study had been previously validated. Validating these measures will be important as further research examines attitudes toward chatbot-delivered mental health support.

## Conclusions

Chatbots continue to gain sophistication in their ability to mimic human communication, and initial data have supported the potential for chatbots to provide mental health support.^5,6^ The results of this study suggest that U.S. students’ utilization of chatbots for mental health support is low and likely to be limited by doubts about helpfulness, including among students with probable depression or anxiety. Nonetheless, the results also highlight the potential for chatbots to reach people who might not otherwise receive traditional mental health services due to time, cost, or stigma concerns. Further characterizing utilization and public opinion of chatbot-delivered support will be essential to inform the design and dissemination of mental health chatbots.

## Supplemental Material

sj-docx-1-dhj-10.1177_20552076241313401 - Supplemental material for Chatbot-delivered mental health support: Attitudes and utilization in a sample of U.S. college students Supplemental material, sj-docx-1-dhj-10.1177_20552076241313401 for Chatbot-delivered mental health support: Attitudes and utilization in a sample of U.S. college students by Gavin N. Rackoff, Zhenyu Z. Zhang and Michelle G. Newman in DIGITAL HEALTH
