# Supplementary material for: Chatbot-delivered mental health support: Attitudes and utilization in a sample of U.S. college students
Source: Digit Health. 2025 Jan 17;11:20552076241313401. doi: 10.1177/20552076241313401 (PMC11748072; doi:10.1177/20552076241313401)
Supplement: sj-docx-1-dhj-10.1177_20552076241313401 - Supplemental material for Chatbot-delivered mental health support: Attitudes and utilization in a sample of U.S. college students [file sj-docx-1-dhj-10.1177_20552076241313401.docx]

**Mental Health Service Utilization and Barriers Assessment**

1. In the**past 12 months**, have you received any treatment for emotional or mental health problems (e.g., therapy, counseling, medication)?

- Yes
- No

1. (For those responding “No” to the previous question): In the**past 12 months**, which of the following explain why you have not received treatment for your mental or emotional health? (Select all that apply)
   - No need for services
   - Financial reasons (too expensive, not covered by insurance)
   - Not enough time
   - Concerns about mental health stigma
   - Don’t think treatment would help
   - Privacy concerns

**Chatbot Utilization and Barriers Assessment**

The next set of questions asks about your experiences and attitudes toward receiving **mental health support from chatbots or digital conversational agents**. By chatbots or digital conversational agents, we are referring to non-human computer programs that are designed to simulate human conversation, including but not limited to programs such as ChatGPT.

1. Have you ever used a chatbot or digital conversational agent for any purpose (even if unrelated to mental health)?

- Yes
- No

1. In the **past 12 months**, have you sought mental health support from a chatbot or digital conversational agent?

- Yes
- No

1. (For those responding “No” to the previous question): In the **past 12 months**, which of the following explain why you have not sought mental health support from a chatbot or digital conversational agent? (Select all that apply)
   - No need for services
   - Financial reasons (too expensive, not covered by insurance)
   - Not enough time
   - Concerns about mental health stigma
   - Don’t think a chatbot or conversational agent would help
   - Privacy concerns

**Mental Help Seeking Attitude Scale–Chatbot Modification (modified version of the validated MHSAS^1^).**

Please mark the circle that best represents your opinion. For example, if you feel that your seeking help would be extremely useless, you would mark the circle closest to "useless." If you are undecided, you would mark the "0" circle. If you feel that your seeking help would be slightly useful, you would mark the "1" circle that is closer to "useful."

If I had a mental health concern, seeking help from a chatbot or digital conversational agent would be...

|  | 3 | 2 | 1 | 0 | 1 | 2 | 3 |  |
| --- | --- | --- | --- | --- | --- | --- | --- | --- |
| Useless | ⃝ | ⃝ | ⃝ | ⃝ | ⃝ | ⃝ | ⃝ | Useful |
| Important | ⃝ | ⃝ | ⃝ | ⃝ | ⃝ | ⃝ | ⃝ | Unimportant |
| Unhealthy | ⃝ | ⃝ | ⃝ | ⃝ | ⃝ | ⃝ | ⃝ | Healthy |
| Ineffective | ⃝ | ⃝ | ⃝ | ⃝ | ⃝ | ⃝ | ⃝ | Effective |
| Good | ⃝ | ⃝ | ⃝ | ⃝ | ⃝ | ⃝ | ⃝ | Bad |
| Healing | ⃝ | ⃝ | ⃝ | ⃝ | ⃝ | ⃝ | ⃝ | Hurting |
| Disempowering | ⃝ | ⃝ | ⃝ | ⃝ | ⃝ | ⃝ | ⃝ | Empowering |
| Satisfying | ⃝ | ⃝ | ⃝ | ⃝ | ⃝ | ⃝ | ⃝ | Unsatisfying |
| Desirable | ⃝ | ⃝ | ⃝ | ⃝ | ⃝ | ⃝ | ⃝ | Undesirable |

^1^ Hammer JH, Parent MC, Spiker DA. Mental Help Seeking Attitudes Scale (MHSAS): Development, reliability, validity, and comparison with the ATSPPH-SF and IASMHS-PO. J Couns Psychol 2018;65(1):74-85, doi:10.1037/cou0000248
